# Comorbidity of Substance Use Disorders and Eating Disorders: a major concern for mental health care professionals

**DOI:** 10.1192/j.eurpsy.2022.1481

**Published:** 2022-09-01

**Authors:** C. Tapoi

**Affiliations:** Prof. Dr. Alexandru Obregia Clinical Psychiatry Hospital, Psychiatry, Bucharest, Romania

**Keywords:** bulimia nervosa, dual diagnosis, eating disorder, substance use disorder

## Abstract

**Introduction:**

During the last 30 years, many studies have shown a high prevalence of substance use among patients diagnosed with an Eating Disorder (ED). Almost 50% of the patients with ED have a history of substance use, and 35% of the patients that seek help for an addiction disorder also meet criteria for ED. Nevertheless, both substance abuse specialists and pratictioners with expertise in ED have difficulties in treating these dually diagnosed patients.

**Objectives:**

The aim of this study is to emphasize the importance of assessing substance use in patients with ED and disturbed eating behaviors in patients with Substance Use Disorders (SUD), as well as the need for evidence-based treatment guidelines for this comorbid condition.

**Methods:**

A literature search of published articles on substance use patterns in ED and on the therapeutic approach for this comorbid condition was performed on PubMed database.

**Results:**

A diagnosis of Bulimia Nervosa and the presence of binging/ purging behaviors are strongly associated with substance use. Most frequently used substances are represented by nicotine, caffeine and alcohol, followed by cannabis and amphetamines. Reasons why patients with ED use substances are emotional regulation and appetite suppression. Detailed and systematic evaluation of the substances used and for other psychiatric comorbidities is mandatory. Management plan involves simultaneously treating ED and SUD.

**Conclusions:**

The comorbidity of Substance Use Disorders and Eating Disorders is a complex entity, but nonetheless treatable. Further studies are needed to specify the patterns of substance use in Eating Disorders and their implications for treatment.

**Disclosure:**

No significant relationships.

